# Disparities in neoadjuvant chemotherapy for pancreatic adenocarcinoma with vascular involvement

**DOI:** 10.1016/j.sopen.2024.06.003

**Published:** 2024-06-18

**Authors:** Nikhil Chervu, Shineui Kim, Sara Sakowitz, Nguyen Le, Saad Mallick, Hanjoo Lee, Peyman Benharash, Timothy Donahue

**Affiliations:** a Department of Surgery, David Geffen School of Medicine, University of California, Los Angeles, CA, USA; b Department of Surgery, Harbor-UCLA Medical Center, Torrance, CA, USA; c Division of Surgical Oncology, Department of Surgery, David Geffen School of Medicine, University of California, Los Angeles, UCLA, Los Angeles, CA, USA

**Keywords:** Pancreatic cancer, Neoadjuvant therapy, Borderline resectable, Locally advanced, Disparities, Pancreatic adenocarcinoma

## Abstract

**Background:**

Multiagent neoadjuvant chemotherapy (NAT) has been linked with improved survival for locally advanced (LA) or borderline resectable (BR) pancreatic ductal adenocarcinoma (PDAC). However, the existence of disparities in its utilization remains to be elucidated.

**Methods:**

All adults with PDAC were tabulated from the 2011–2017 Nationwide Cancer Database. Tumor vascular involvement was determined using the clinical T stage and CS_EXTENSION variables. The significance of temporal trends was calculated using Cuzick's non-parametric test. A Cox proportional hazard model was used to assess the impact of NAT utilization on hazard of two-year mortality. A logistic regression model was developed to determine factors associated with receipt of NAT.

**Results:**

Of 3811 patients meeting inclusion criteria, 50.8 % received NAT. NAT utilization significantly increased over the study period, from 31.7 % in 2011 to 81.1 % in 2017 (*p* < 0.001). NAT was associated with significantly reduced two-year mortality (Hazards Ratio 0.34, 95 % Confidence Interval [CI] 0.18–0.67).

After adjustment, younger (Adjusted Odds Ratio [AOR] 0.97/year, CI 0.96–0.98) and Black (AOR 0.65, CI 0.48–0.89; ref: White) patients demonstrated reduced odds of NAT. Furthermore, patients with Medicare (AOR 0.73, CI 0.59–0.90; ref: Private) or Medicaid insurance (AOR 0.67, CI 0.46–0.97; ref: Private) had lower odds of NAT, as did those treated at non-academic institutions (Community: AOR 0.42, CI 0.35–0.52, Integrated: 0.68, CI 0.54–0.85) or in the lowest education quartile (AOR 0.52, CI 0.29–0.95; ref: Highest).

**Conclusions:**

We identified increasing utilization of NAT for BR/LA pancreatic adenocarcinoma. Despite being linked with significantly reduced two-year mortality, socioeconomic disparities affect odds of NAT.

## Introduction

With an estimated 5-year survival rate of <10 %, pancreatic ductal adenocarcinoma (PDAC) is the third leading cause of cancer-related deaths in the United States [1,2]. The inherent aggressiveness of PDAC frequently results in patients presenting with locally advanced (LA) or borderline resectable (BR) disease. Neoadjuvant chemotherapy (NAT) has therefore been introduced to improve surgical resection rates and long-term survival [3]. However, few studies have examined disparities in access to NAT at a large-scale.

Traditionally, the primary curative treatment for localized PDAC has entailed upfront resection followed by adjuvant chemotherapy [4,5]. Even among patients who successfully undergo pancreatic resection and subsequent adjuvant therapy, however, early recurrences are common, resulting in an overall survival of <20 months [6]. NAT aims to improve resectability by downstaging tumors, enabling margin-negative (R0) resection, and converting initially unresectable tumors into operable ones [7,8]. Prior work by Cloyd et al. has demonstrated that uninsured and Medicaid patients have lower odds of receiving NAT in Stage I and II PDAC [9]. Nevertheless, limited data exists specifically for such disparities in BR and LA PDAC, for which NAT was initially described.

We used a large nationwide database to retrospectively analyze disparities in receipt of NAT for LA and BR PDAC. We hypothesize that tumor-independent socioeconomic factors would be associated with reduced utilization of NAT for PDAC, despite improvement in survival benefit.

## Methods

**Fig. 1:**
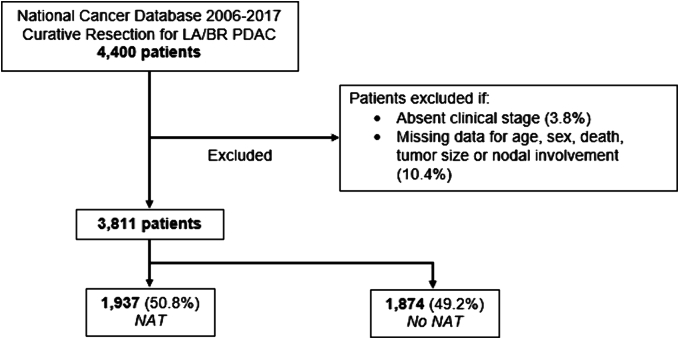
CONSORT diagram with survey-weighted estimates; LA, locally advanced; BR, borderline resectable; PDAC, pancreatic ductal adenocarcinoma; NAT, neoadjuvant chemotherapy.

All adults (≥18 years) who underwent curative-intent neoadjuvant chemotherapy or upfront resection for locally advanced or borderline resectable pancreatic ductal adenocarcinoma were identified within the 2011–2017 Nationwide Cancer Database (NCDB). Jointly managed by the American College of Surgeons and American Cancer Society, the NCDB captures nearly 70 % of all newly diagnosed cancer patients in the United States. Patient data is collected from all cancer patients seen at Commission on Cancer (COC) sites [10]. Tumor vascular involvement was determined using the clinical T stage and CS_EXTENSION variables. Specifically, all patients with clinical T4 stage disease were deemed to have LA disease. Those with other arterial or venous involvement based on CS_EXTENSION codes were otherwise classified as borderline resectable (BR), as previously published by Chawla et al. [11] Patients with missing data for age, sex, death, tumor size, clinical stage, or nodal involvement were excluded from analysis (14.2 %; Fig. 1).

The NCDB data dictionary was used to define additional patient, hospital, and tumor characteristics. Variables included age, race, income, insurance status, clinical and pathological T, N, and M stage, tumor size, surgical intervention, and neoadjuvant or adjuvant chemoradiation. Multiple revisions of the American Joint Committee on Cancer's Staging System were present throughout the study period. Tumor stage was updated to the 8th edition using the CS_EXTENSION variable, tumor size, T, N, and M stage [10,12]. The Charlson-Deyo score was used to account for comorbid conditions in risk-adjusted analyses [13]. Patients who received curative-intent neoadjuvant chemotherapy with or without subsequent surgical resection were considered to have had NAT for an intention-to-treat analysis.

Continuous variables are reported as means with standard deviation (SD), or as medians with interquartile range (IQR) if not normally distributed. Categorical variables are presented as frequencies (%). The Welch's *t*-test of unequal variance and Pearson's χ^2^ tests were used to determine the significance of intergroup differences for continuous and categorical variables, respectively. Cuzick's non-parametric rank-based test was used to assess the statistical significance of temporal trends (*nptrend*) [14]. A Cox proportional hazard model was then used to assess the impact of NAT utilization on hazard of two-year mortality. Subsequently, a multivariable logistic regression model was developed to determine factors associated with patient, tumor, and hospital factors associated with receipt of NAT. Regression outputs are reported as Adjusted Odds Ratios (AOR) with 95 % Confidence Intervals (CI). α of 0.05 was set for statistical significance. Given our larger sample size, we used standardized mean difference (SMD) to assess effect size with a value >0.10 considered significant. All statistical analyses were performed using Stata 16 (StataCorp, College Station, TX).

Data from the NCDB is deidentified and HIPAA compliant, with no attempt to contact or identify subjects by the authors. This study was deemed exempt from full review by the Institutional Review Board at the University of California, Los Angeles.

## Results

**Fig. 2:**
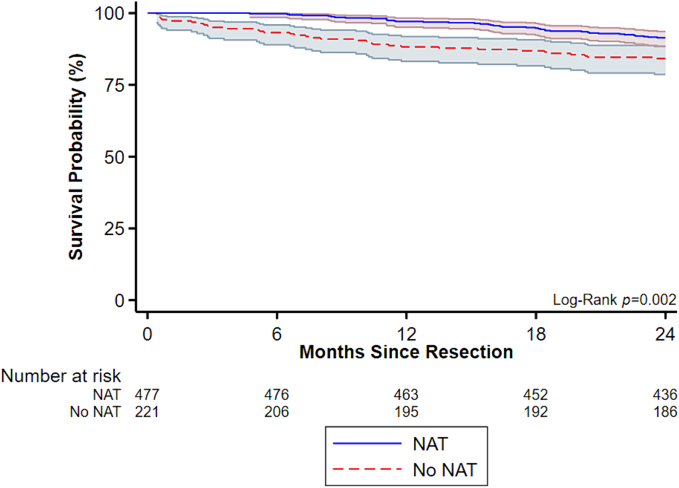
Kaplan-Meier two-year survival estimates of patients receiving multiagent neoadjuvant chemotherapy (NAT) for pancreatic ductal adenocarcinoma.

Of an estimated 3811 patients, 50.8 % (*n* = 1937) received NAT for LA/BR disease. Throughout the study period, NAT utilization significantly increased from 31.3 % in 2011 to 81.1 % in 2017 (*nptrend* < 0.001). Compared to others, NAT patients were younger (62.8 years ± 9.4 vs 66.3 ± 10.0, *p* < 0.001, SMD = 0.37) and more commonly White (88.3 vs 86.6 %, *p* = 0.018, SMD < 0.01). Patients receiving NAT more commonly had private insurance (49.0 vs 34.7 %, *p* < 0.001, SMD = 0.02) and were more frequently managed at academic centers (63.9 vs 48.8 %, *p* < 0.001, SMD = 0.06) in the Northeastern (23.9 vs 20.4 %, *p* = 0.005, SMD = 0.02) and Western (18.0 vs 16.9 %, *p* = 0.005, SMD = 0.02) regions, compared to their non-NAT counterparts. Finally, patients who had NAT were more likely to have LA tumors (56.3 vs 42.6 %, *p* < 0.001, SMD = 0.12) located in the body of the pancreas (15.2 vs 10.9 %, *p* < 0.001, SMD < 0.01; Table 1). Cox proportional hazard modeling demonstrated that NAT was associated with significantly reduced two-year mortality (Hazard Ratio 0.34, CI 0.18–0.67, reference: no NAT; Fig. 2).

**Table 1:** Patient, clinical, and hospital characteristics of patients diagnosed with curative resection for pancreatic ductal adenocarcinoma with or without neoadjuvant chemotherapy (NAT); SMD, standardized mean difference; SD, standard deviation; IQR, interquartile range; mm, millimeter.

	NAT (n = 1937)	No NAT (*n* = 1874)	*p*-Value	SMD
Patient characteristics				
Age (years, mean ± SD)	62.8 ± 9.4	66.3 ± 10.0	<0.001	0.37
Female (%)	971 (50.1)	927 (49.5)	0.68	0.01
Race (%)			0.018	<0.01
White	1711 (88.3)	1623 (86.6)		
Black	131 (6.8)	176 (9.4)		
Asian/Pacific Islander	55 (2.8)	44 (2.3)		
Other/unspecified	40 (2.1)	31 (1.7)		
Income quartile (%)			<0.001	0.02
76th–100th	1338 (69.1)	1346 (71.8)		
51st–75th	113 (5.8)	101 (5.4)		
26th–50th	98 (5.1)	105 (5.6)		
0–25th	99 (5.1)	126 (6.7)		
Primary payer (%)			<0.001	0.02
Private	950 (49.0)	650 (34.7)		
Medicare	802 (41.4)	1061 (56.6)		
Medicaid	95 (4.9)	97 (5.2)		
Uninsured	26 (1.3)	31 (1.7)		
Other/unspecified	64 (3.3)	35 (1.9)		
Education quartile (%)			<0.001	0.02
76th–100th	1542 (79.6)	1557 (83.1)		
51st–75th	46 (2.4)	44 (2.4)		
26th–50th	38 (2.0)	29 (1.6)		
0–25th	22 (1.1)	48 (2.6)		
Charlson-Deyo Score (%)			0.12	0.01
0	1335 (68.9)	1247 (66.5)		
1	477 (24.6)	477 (25.5)		
≥2	125 (6.5)	150 (8.0)		

Tumor characteristics				
Tumor size (mm, mean ± SD)	36.6 ± 28.6	38.6 ± 19.8	0.014	0.08
Tumor location (%)			<0.001	<0.01
Head	1327 (68.5)	1309 (69.9)		
Body	294 (15.2)	204 (10.9)		
Tail	126 (6.5)	230 (12.3)		
Other/unspecified	190 (9.8)	131 (7.0)		
Nodal involvement (%)	612 (31.6)	514 (27.4)	0.005	0.04
Resectable status (%)			<0.001	0.12
Locally advanced	1090 (56.3)	799 (42.6)		
Borderline resectable	847 (43.7)	1075 (57.4)		
Grade (%)			<0.001	0.55
Well	117 (6.0)	139 (7.4)		
Moderate	516 (26.6)	769 (41.0)		
Poor/undifferentiated	322 (16.6)	611 (32.6)		
Unknown	982 (50.7)	355 (18.9)		

Hospital characteristics				
Hospital region (%)			0.005	0.02
Northeast	462 (23.9)	382 (20.4)		
Midwest	531 (27.4)	521 (27.8)		
South	575 (29.7)	643 (34.3)		
West	348 (18.0)	317 (16.9)		
Hospital type (%)			<0.001	0.06
Academic	1238 (63.9)	915 (48.8)		
Community	382 (19.7)	592 (31.6)		
Integrated network	296 (15.3)	356 (19.0)

**Fig. 3:**
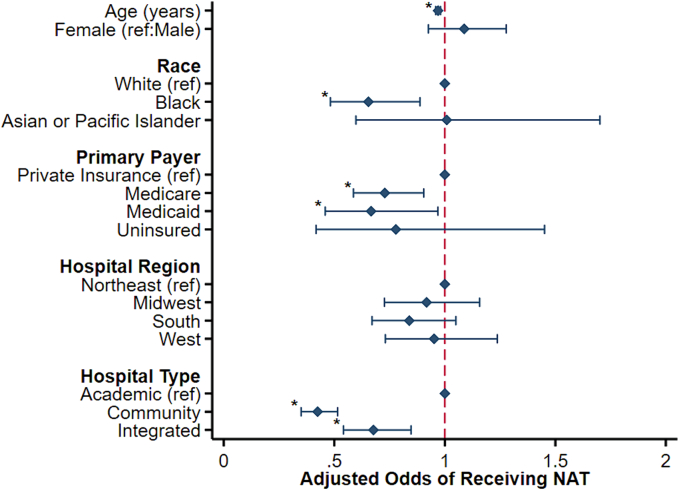
Demographic, socioeconomic, and hospital characteristics associated with altered odds of receiving NAT for pancreatic ductal adenocarcinoma; *p < 0.05.

After adjustment, several non-tumor factors were associated with receipt of NAT (Table 2). Younger (AOR 0.97/year, CI 0.96–0.98) and Black (AOR 0.65, CI 0.48–0.89; ref: White) patients demonstrated reduced odds of NAT. Furthermore, patients with Medicare (AOR 0.73, CI 0.59–0.90; ref: Private) or Medicaid insurance (AOR 0.67, CI 0.46–0.97; ref: Private) had lower odds of NAT, as did those in the lowest education quartile (AOR 0.52, CI 0.29–0.95; ref: Highest). Patients treated at non-academic centers (Community: AOR 0.42, CI 0.35–0.52 and Integrated: 0.68, CI 0.54–0.85) also had reduced odds of NAT (Fig. 3).

**Table 2:** Factors associated with receipt of neoadjuvant chemotherapy (NAT) in the treatment of locally advanced (LA) or borderline resectable (BR) pancreatic ductal adenocarcinoma; AOR, adjusted odds ratio; CI, confidence interval; REF, reference.

	AOR	95 % CI	p-Value
Patient characteristics			
Age (per year)	0.97	0.96–0.98	<0.001
Female	1.08	0.93–1.28	0.31
Race			
White	REF	–	–
Black	0.65	0.48–0.89	0.006
Asian/Pacific Islander	1.01	0.60–1.70	0.98
Other/unspecified	0.77	0.37–1.60	0.49
Income quartile			
76th–100th	REF	–	–
51st–75th	1.07	0.77–1.50	0.67
26th–50th	0.88	0.63–1.24	0.47
0–25th	0.86	0.62–1.19	0.36
Primary payer			
Private	REF	–	–
Medicare	0.73	0.59–0.90	0.004
Medicaid	0.67	0.46–0.97	0.033
Uninsured	0.78	0.42–1.45	0.43
Other/unspecified	1.49	0.73–3.03	0.28
Education quartile			
76th–100th	REF	–	–
51st–75th	1.08	0.66–1.78	0.76
26th–50th	1.64	0.93–2.89	0.087
0–25th	0.52	0.29–0.95	0.033
Charlson-Deyo Score (%)			
0	REF	–	–
1	0.99	0.82–1.19	0.89
≥2	0.81	0.59–1.12	0.20

Tumor characteristics			
Tumor size (per mm)	1.00	0.99–1.00	0.32
Tumor location (%)			
Head	REF	–	–
Body	1.15	0.89–1.47	0.28
Tail	0.60	0.45–0.81	0.001
Other/unspecified	0.94	0.68–1.29	0.70
Nodal involvement (%)	1.20	1.01–1.44	0.040
Resectable status (%)			
Locally advanced	REF	–	–
Borderline resectable	0.81	0.68–0.98	0.029

Hospital characteristics			
Hospital region (%)			
Northeast	REF	–	–
Midwest	0.92	0.73–1.16	0.47
South	0.84	0.67–1.05	0.13
West	0.95	0.73–1.24	0.71
Hospital type (%)			
Academic	REF	–	–
Community	0.42	0.35–0.52	<0.001
Integrated network	0.68	0.54–0.85	0.001

## Discussion

Since its introduction in the 1950s, NAT has been used in a variety of cancers to improve surgical resectability and reduce systemic disease burden to prolong survival [15]. For patients with PDAC, it may provide a chance to complete a full treatment of multiagent chemotherapy, as many patients are too frail postoperatively to undergo scheduled adjuvant therapy [9]. Our results demonstrate that, despite increasing utilization and improved two-year survival, there exist significant demographic and socioeconomic disparities in the receipt of NAT independent of tumor factors. Specifically, Black patients and those with Medicare or Medicaid insurance had lower odds of NAT, compared to their White or privately insured counterparts. Furthermore, patients treated at non-academic institutions were less likely to undergo NAT prior to curative resection. These findings warrant further discussion.

We found that NAT utilization increased 5-fold throughout the study period and was associated with an 66 % reduction in hazard of two-year mortality for LA/BR PDAC. Older studies examining resectable as well as LA/BR PDAC have similarly found increased utilization of NAT [16,17]. In combination with improved survival, current National Comprehensive Cancer Network (NCCN) guidelines thus recommend NAT in all cases of LA and BR PDAC [18]. Specifically, complete pathologic response to NAT was shown to increase median overall survival from 23 months to 43 months in a single-institution study [19]. Complete pathologic response notwithstanding, NAT has also been associated with lower rates of recurrence [20,21]. Despite these updated guidelines, it should be noted that the PREOPANC-1 trial is the only prospective study proving improved survival in resectable and BR pancreatic cancer patients [22]. Although a discussion of specific regimens is out of the scope of this manuscript, trials examining PEXG, gemcitabine, and FOLFIRINOX are currently ongoing and may further determine benefits to survival and resectability [[23], [24], [25]]. As novel regimens and therapeutic combinations continue to be tested, future studies examining their efficacy at a large-scale will be necessary.

In spite of improved survival, significant racial and insurance disparities exist in receipt of NAT for LA/BR PDAC. In this cohort, Black patients had lower odds than White patients of undergoing NAT prior to curative resection. Black patients have been shown to have significantly lower pathologic complete response to NAT and quality of care for a variety of oncologic conditions, including bladder, ovarian, and breast cancer [[26], [27], [28]]. Nevertheless, many of these of studies show increased utilization of NAT in Black patients. A 2020 study by Cloyd et al. similarly showed no significant difference in NAT utilization between Black and White patients with Stage I-II, mainly resectable, PDAC [9]. Our findings, therefore, may represent a previously unrecognized disparity within pancreatic cancer care. These disparities can be recognized when looking at other aspects of pancreatic cancer in the Black population. Black patients have a higher incidence of and mortality due to PDAC as well as presenting at later stages, compared to White patients [[29], [30], [31]]. Moreover, a 2013 study by Shah et al. found that pancreatic cancer resection was less often recommended to and performed for Black patients [32]. We likewise found that patients with Medicare or Medicaid insurance had lower odds of NAT utilization, which reflects findings by other authors [31,[33], [34], [35]]. Improved insurance coverage has already been demonstrated to improve pancreatic cancer care. Loehrer et al. demonstrated improved resection rates for patients in Massachusetts with government-subsidized insurance after 2006 Medicaid expansion [36]. As revised treatment strategies continue to improve survival and quality of life, addressing racial and insurance disparities must be addressed to improve overall healthcare.

Our results indicate that non-academic institutions were also associated with reduced odds of NAT for patients with LA/BR PDAC. Non-academic centers have been associated with reduced uptake of neoadjuvant therapy across a spectrum of cancers [37,38]. This may due to differences in annual volumes. As a result of the overall low prevalence of pancreatic cancer, high volume operative centers tend to be academic institutions with lower surgical refusal rates than at non-academic institutions [39,40]. Unfortunately, rural patients as well as those who are of older, female, of black race, and uninsured have been shown to have increased refusal rates even at academic centers [40]. Although we, and other groups, adjust for certain demographic and socioeconomic factors, our results likely highlight reduced access to comprehensive care options.

Several limitations are present inherent to the retrospective study design and use of an administrative database. The NCDB only collects data from COC sites, and as such, patients with historically poor access to healthcare may not be accounted for. While the NCDB has significant detail regarding tumor staging and histology, it does not contain information from physician-patient encounters or intraoperative data that may affect overall results. Although information regarding time from diagnosis to start of chemotherapy is available, details such as duration of treatment, specific agents, and cycle specifics are unavailable. As a result, we were unable to incorporate patients who had upfront chemotherapy without surgery due to an inability to adjust for lead-time bias. Finally, we are unable to draw any causal relationships.

In conclusion, despite improved survival and incorporation into current NCCN guidelines, NAT usage in the treatment of LA/BR PDAC is subject to significant non-clinical disparities. Specifically, racial, insurance-based, and center-level differences in the receipt of NAT must be addressed to ensure equitable cancer care nationwide. Future work should be directed at systems to enable patients access to high-volume, operative, and multidisciplinary cancer centers.

## Funding/financial support

No direct or indirect financial support by extramural sources was received for this research.

## Ethics approval

This manuscript use deidentified data from a publicly available administrative database and was thus deemed exempt from full review by the University of California, Los Angeles Institutional Review Board.

## CRediT authorship contribution statement

**Nikhil Chervu:** Conceptualization, Data curation, Formal analysis, Investigation, Methodology, Project administration, Resources, Validation, Visualization, Writing – original draft, Writing – review & editing. **Shineui Kim:** Conceptualization, Methodology, Writing – original draft, Writing – review & editing. **Sara Sakowitz:** Conceptualization, Data curation, Formal analysis, Methodology, Writing – review & editing. **Nguyen Le:** Conceptualization, Methodology, Validation, Writing – original draft. **Saad Mallick:** Conceptualization, Data curation, Methodology, Writing – review & editing. **Hanjoo Lee:** Data curation, Resources. **Peyman Benharash:** Conceptualization, Project administration, Validation, Visualization, Writing – review & editing. **Timothy Donahue:** Conceptualization, Supervision, Validation, Writing – review & editing.

## Declaration of competing interest

Dr. Peyman Benharash received fees from AtriCure as a surgical proctor. This manuscript does not discuss any AtriCure products or services. Other authors report no conflicts.
